# Complex Management of AV Nodal Agent Toxicity in Patients with Cardiac Devices: Massive Calcium Channel Antagonist Overdose in a Patient with a Permanent Pacemaker

**Published:** 2022-01-27

**Authors:** Patric W. Gibbons, Peter R. Chai, Timothy B. Erickson

**Affiliations:** 1 Department of Emergency Medicine, Division of Medical Toxicology, Brigham and Women’s Hospital, Massachusetts General Hospital, Boston, USA; 2 Department of Psychosocial Oncology and Palliative Care, Dana Farber Cancer Institute, Boston, USA; 3 The Koch Institute for Integrated Cancer Research, Massachusetts Institute of Technology, Cambridge, USA; 4 The Fenway Institute, Boston, USA; 5 Harvard Humanitarian Institute, Cambridge, USA

**Keywords:** calcium channel blocker overdose, beta-blocker overdose, nodal toxicity, pacemaker

## Abstract

We present a unique case of a massive calcium channel antagonist overdose in a patient with a permanent pacemaker. Upon presentation after the acute overdose, the patient’s cardiac device was found to be pacing to an adequate rate (75 beats per minute) and she was admitted to the cardiac intensive care unit. Approximately 24 hours after her ingestion, she acutely decompensated and became hypotensive. The patient was started on infusions of norepinephrine, epinephrine, phenylephrine, and vasopressin. Her mean arterial pressure was unresponsive to multi-vasopressor therapy. She was then given a bolus of methylene blue and high-dose insulin euglycemic therapy. Despite these treatments, the patient remained hypotensive Therefore, intralipid emulsion therapy and IV epinephrine pushes were also administered. As a result of her shock and hemodynamic instability, her course was further complicated by hypoxemic respiratory failure for which she required ventilatory support and developed oliguric renal failure for which she was initiated on continuous veno-venous hemofiltration. This case emphasizes the challenges in managing complex physiology associated with nodal agent toxicity and is the first, to our knowledge, to describe management in a patient who already had a pacemaker, though it was ultimately ineffective in avoiding the patient’s profound decompensation.

## Introduction

Calcium channel and beta receptor antagonists are common xenobiotics used to manage hypertension and various supraventricular arrythmias. In toxic doses, these agents present challenging metabolic and physiological derangements for the clinician to manage. Symptomatic bradycardia is a hallmark feature of nodal agent toxicity. This bradycardia may be unresponsive to typical pharmacotherapy like atropine. An alternative strategy to manage these poisoned individuals may be to utilize transcutaneous or transvenous pacing as a temporalizing measure until drug toxicity wanes [1]. One important population who are frequently prescribed nodal blocking agents are those with preexisting implantable cardiac devices like pacemakers or left ventricular assist devices (LVADs). Nodal poisoning in these patients is less well described. We present a case of a massive calcium channel antagonist overdose in a patient with a permanent pacemaker.

## Case report

A 30-year-old female with a history of congenital long QT syndrome with a dual automated implantable cardioverter-defibrillator (AICD)/pacemaker presented to the emergency department (ED) after ingesting approximately 90 tablets of 120mg extended-release diltiazem (103mg/kg) approximately one hour prior. She arrived via private vehicle at the ED hemodynamically stable, with a heart rate of 79beats per minute and a blood pressure of 101/53 mmHg. Initial finger stick glucose was 97 mg/dL. Her triage electrocardiogram (ECG) (Figure 1a) showed a Ventricular-paced rhythm with a prolonged PR interval (280 ms). A baseline ECG from several weeks prior (Figure 1b) demonstrated that these changes were new, suggesting nodal blockade from intentional diltiazem ingestion. On physical examination, she was fully alert and oriented and denied any medical complaints including chest pain, shortness of breath, dizziness, lightheadedness or syncope. Lung sounds were clear, heart sounds were normal, and patient had 2+ peripheral pulses. Laboratory studies were generally unrevealing including normal serum electrolyte levels, a negative urine toxicology panel, and undetected salicylates, tricyclics, ethanol and acetaminophen levels.

Since her cardiac device was found to be pacing to an adequate rate (75 beats per minute) and she remained hemodynamically stable, standard therapies such as pressor agent administration or high-dose insulin euglycemic therapy (HIET) were held and supportive care administered. She was admitted to the cardiac intensive care unit (CCU) where an arterial line was placed, and she was monitored overnight. Approximately 24 hours after her ingestion, she acutely decompensated after a near syncopal event with urination. She was found hypotensive, and a right internal jugular central line was emergently placed. She was subsequently started on infusions of norepinephrine at 30mcg/min, epinephrine at 20 mcg/min, phenylephrine at 200mcg/min and vasopressin at 0.1U/min. Despite multi-vasopressor therapy, her mean arterial pressure remained in the 50-60 range. She was then administered a bolus of methylene blue (1.5mg/kg) and HIE was initiated at 1U/kg bolus titrated to 1U/kg/hr. Despite these treatments, it remained challenging for the CCU team to maintain her mean arterial pressure (MAP) greater than 60 mmHg. Therefore, 100mg intralipid was given and five 0.5mg IV epinephrine pushes were also administered. Over the next 24 hours, her shock gradually resolved, and vasopressors were weaned. As a result of her shock and hemodynamic instability, her course was further complicated by hypoxemic respiratory failure for which she was orally intubated (using succinylcholine and propofol) and oliguric renal failure for which she was initiated on continuous veno-venous hemofiltration (CVVH). On hospital day 12, she was successfully extubated. She was discharged from the CCU on hospital day 17 and ultimately discharged from the hospital after a 27 day stay and was reportedly doing well overall with good renal recovery.

## Discussion

This case emphasizes the challenges in managing complex physiology associated with nodal agent toxicity and is the first, to our knowledge, to describe management in a patient who already had a pacemaker, though it was ultimately ineffective in avoiding the patient’s profound decompensation. Furthermore, this case highlights the multiple mechanisms through which nodal agents exert their toxicity, most notably inotropy, chronotropy, and vascular resistance. In pacemaker cells, nodal agents prolong phase 0 of the action potential, leading to decreased heart rate [1]. Previous case reports have shown some success with transvenous pacing in improving heart rate and blood pressure in the setting of nodal agent overdose [2-4]. However, in cardiac myocytes, nodal agents will decrease the influx of calcium into the sarcoplasmic reticulum, thereby reducing the strength of contraction leading to cardiogenic shock. Calcium channel blockers will also decrease after load, leading to vasodilatory shock [1]. As such, a pacemaker will likely be ineffective in correcting these other, non-chronotropic mechanisms. Cardiac pacing may be effective in increasing the rate of myocardial contraction however, electrical capture is not always successful and adequate blood pressure may not be restored [5]. It is not known how nodal agent toxicity will behave in the setting of an LVAD, though a handful of case reports described successful resuscitation of patients using percutaneous left ventricular assist (Impella) heart pump devices [6-8]. This is an interesting area of future research as one study has shown increased rates of suicide attempts in patients with LVADs. [9].

## Conclusion

The clinical state of a patient poisoned with nodal agents is not solely dependent on their heart rate. As shown in this case, patients with stable initial vital signs may decompensate quickly, particularly with extended-release prepartions. Standard interventions including pressor agent administration and HIET should be considered and with intensive care monitoring even in the presence of a pacemaker device.

## Figures and Tables

**Figure 1a.**
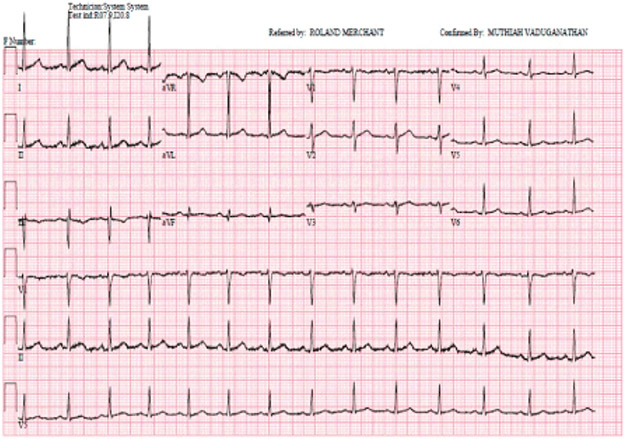
Triage EKG upon arrival to the Emergency Department

**Figure 1b.**
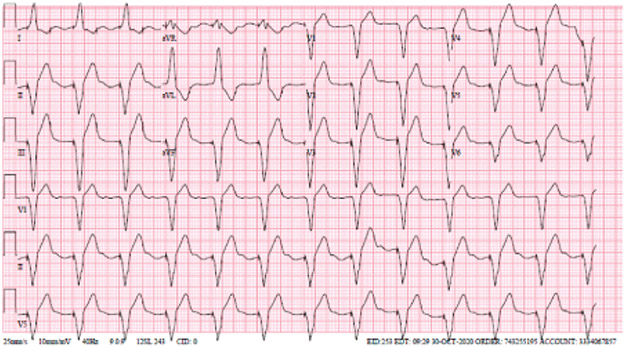
Patient’s Baseline EKG from a routine outpatient visit several weeks prior
